# Expansion of the Phenotypic and Genotypic Spectrum of MED13L‐Associated Neurodevelopmental Disorder: A Case Report and Literature Review

**DOI:** 10.1002/mgg3.70254

**Published:** 2026-06-25

**Authors:** Zhongqing Wang, Xin Chen, Ziyun Cui, Xinyue Qi, Li Gu, Yi Liu, Wenjing Zhao, Yan Jiang

**Affiliations:** ^1^ Medical Genetics Department of the First People's Hospital of Yunnan Province/Affiliated Hospital of Kunming University of Science and Technology, NHC Key Laboratory of Healthy Birth and Birth Defect Prevention in Western China, Yunnan Provincial Key Laboratory for Birth Defects and Genetic Diseases The First People's Hospital of Yunnan Province Kunming Yunnan China; ^2^ School of Medicine Kunming University of Science and Technology Kunming Yunnan China; ^3^ Department of Medical Genetics Yunnan Maternal and Child Health Hospital Kunming Yunnan China; ^4^ Department of Pediatrics Yunnan First People's Hospital Kunming Yunnan China; ^5^ Preventive Health Care Department First People's Hospital of Yunnan Province Kunming Yunnan China; ^6^ Department of Medical Genetics, NHC Key Laboratory of Healthy Birth and Birth Defect Prevention in Western China, Yunnan Provincial Key Laboratory for Birth Defects and Genetic Diseases The First People's Hospital of Yunnan Province Kunming Yunnan China

**Keywords:** congenital heart defect, copy number changes, intellectual disability, MED13L

## Abstract

**Background:**

Pathogenic variants in *MED13L*, including copy‐number changes and sequence variants, cause *MED13L* syndrome. This rare neurodevelopmental disorder is characterized by global developmental delay, intellectual disability (ID), distinctive facial dysmorphism, hypotonia, and variable congenital heart defects. The phenotypic heterogeneity of *MED13L* syndrome underscores the significance of genotype–phenotype correlation analysis for improving clinical diagnosis and precise phenotypic characterization of affected individuals.

**Methods:**

Venous blood samples anticoagulated with EDTA were collected from the patient and family members, followed by whole‐exome sequencing analysis and subsequent validation using fluorescent quantitative PCR. Concurrently, a systematic search of the PubMed database was performed to summarize previously reported cases harboring *MED13L* copy number Variations (CNVs).

**Results:**

Whole‐exome sequencing revealed a de novo heterozygous single‐copy duplication of a ~19‐kb region within the *MED13L* gene (exons 8–16) in the proband. The patient presents phenotypic features consistent with *MED13L* syndrome and represents the first reported case exhibiting cleft lip. A literature review indicates that vision impairment, developmental delay, and congenital heart disease are widely observed phenotypes, while other less frequent manifestations vary between patients with duplications and deletions.

**Conclusion:**

This study, integrating a case report and a literature review, provides important reference evidence for the clinical genetic counseling of *MED13L* syndrome.

## Graphical abstract

Here we report a novel de novo duplication of exons 8–16 of the MED13L gene in a patient with MED13L syndrome, presenting with an unreported phenotype: cleft lip. A review of previously reported patients with MED13L copy number variants is also conducted to refine genotype–phenotype correlations.

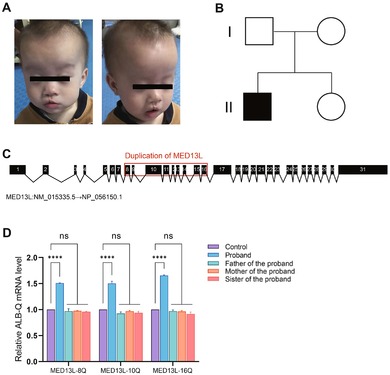

## 1 Introduction

The *MED13L* gene encodes a subunit of the CDK8‐associated mediator complex, which plays a crucial role in transcriptional regulation by bridging DNA‐binding transcription factors and RNA polymerase II (Malik and Roeder 2010). It has been implicated in both congenital heart defects and neurodevelopmental disorders. In 2003, three missense variants in *MED13L* were first identified in a patient with transposition of the great arteries (TGA) (Muncke et al. 2003). In 2013, Asadollahi et al. first described *MED13L* haploinsufficiency syndrome, characterized by intellectual disability, speech and motor delays, and dysmorphic features (Asadollahi et al. 2013). In 2015, Adegbola et al. proposed the term “*MED13L* syndrome” to encompass both truncating variants and CNVs affecting the gene (Meng et al. 2017). The core clinical features include developmental delay, speech delay, ID, characteristic facial appearance (e.g., upslanted palpebral fissures, broad nasal tip, wide mouth), and variable congenital heart defects with incomplete penetrance (Cafiero et al. 2015; Asadollahi et al. 2017; Mitchel et al. 2024). Functional studies have demonstrated that *MED13L* plays a critical role in neurogenesis and neural crest cell development (Asadollahi et al. 2017). This syndrome is uncommon, and previous reports have documented both copy number variants and single‐nucleotide variants in the *MED13L* gene.

Here, we report a patient with a de novo partial duplication of *MED13L* exons 8–16, presenting with global developmental delay, ASD, dysmorphic facial features, and cleft lip. Notably, this is the first reported individual with cleft lip, highlighting the variable expressivity of *MED13L*‐related disorders. We summarize the clinical and genetic findings of all published *MED13L* CNV cases to provide a reference for diagnosis and management.

## 2 Methods

### 2.1 Genetic Testing and Analysis

With informed consent from the patient's family, 2 mL of EDTA‐anticoagulated venous blood was taken from the child and the parents, and the whole exome sequencing (WES) technique was used for testing. Using the single‐copy ALB gene as an internal reference gene, fluorescent quantitative PCR was employed to detect and validate the copy numbers of exons 8, 10, and 16 of the external target gene *MED13L* in normal samples and samples from members of this family.

### 2.2 Literature Collection

The Biomedical Literature Database (PubMed) was searched. The search time limit, using “*MED13L* syndrome” and “*MED13L*” as keywords, was from the creation of the database up to December 2025. Related data were reviewed.

## 3 Results

### 3.1 Case Description

The proband was a 16‐month‐old male infant who exhibited speech and motor delay. He was born at term by cesarean section, the second child of healthy, non‐consanguineous parents, and he had one healthy sister. The patient could sit normally and stand with support, but was unable to crawl fully, could not stand alone, showed reduced manual dexterity, and had a slow reaction time. He produced a small number of non‐meaningful single sounds, did not imitate sounds, gestures, or movements, and was unable to identify facial features or simple objects.

**FIGURE 1:**
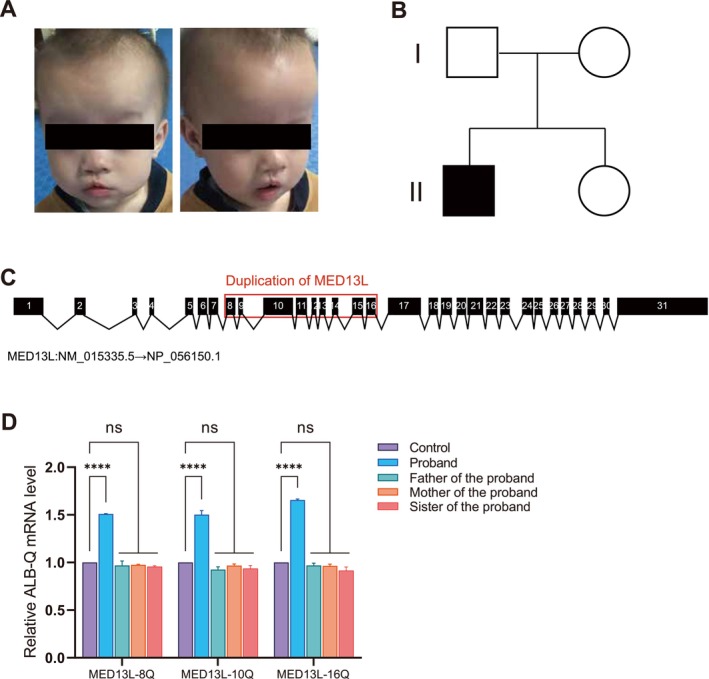
(A) Clinical photographs of the patient showing craniofacial features, including a broad forehead, short palpebral fissures, a wide nasal bridge, a large open mouth with macroglossia and cleft lip, low‐set ears, and a preauricular skin tag on the right side. (B) Pedigree chart of the family. (C) Schematic representation of the *MED13L* gene with a duplication involving exons 8–16. (D) q‐PCR analysis revealed significantly elevated copy number of *MED13L* exons 8, 10, and 16 in the patient compared with other family members.

Dysmorphic facial features included a broad forehead, short palpebral fissures, a wide nasal bridge, a large open mouth, macroglossia, cleft lip, low‐set ears, and a preauricular skin tag on the right side (Figure 1A). Neurological examination revealed unstable muscle tone with episodes of spasms and limb flexion in response to nervousness.

Physical examination revealed micropenis and cryptorchidism, for which he was receiving chorionic gonadotrophin therapy. He also had congenital heart disease—an atrial septal defect—that was surgically treated with defect occlusion in May 2019.

Cranial MRI could not be performed due to metallic interference from the cardiac occlusion device; however, the cranial CT revealed mild widening of the bilateral frontal extracerebral space. Electroencephalography during wakefulness showed increased δ and θ activity. Visual acuity testing revealed mild astigmatism, and CT imaging demonstrated bilateral tympanic membrane thickening. Auditory brainstem response (ABR) testing indicated mild bilateral hearing impairment in both ears (R50 dBnHL, L50 dBnHL). Gesell developmental assessment demonstrated moderate‐to‐severe global developmental delay with deficits across all functional domains; the electrocardiogram, hip X‐ray, and bone mineral density were normal, and chromosome karyotype analysis showed no significant abnormalities.

### 3.2 Genetic Analyses

Pedigree analysis revealed that the proband was the only family member with a partial exon duplication of *MED13L*. The proband's parents and sister were genetically normal (Figure 1B). Whole‐exome sequencing (WES) revealed a heterozygous duplication of exons 8–16 of the *MED13L* gene in the proband (Figure 1C). The expression levels of *MED13L* exons 8, 10, and 16 in the proband were significantly elevated compared to the ALB reference gene, whereas parental and sister samples showed normal expression levels. (Figure 1D). These findings confirm that the *MED13L* copy‐number duplication occurred de novo in the proband.

### 3.3 Review of the Literature

A total of 33 patients with *MED13L* gene variants were identified from 10 publications, including 6 patients with duplication variants and 27 patients with deletion variants (Table 1). These patients exhibited some similar facial dysmorphism and clinical symptoms. However, the total number of patients available for analysis varies across different phenotypes, as not all publications reported complete data for every clinical feature.

**TABLE 1:** Phenotypes of patients with *MED13L* variants. Note: NA, not available; del, deletion; dup, duplication; ID/DD, intellectual disability/developmental delay; TOF, tetralogy of fallot; VSD, ventricular septal defect; PFO, patent foramen ovale; ASD, atrial septal defects.

PMID	This study	23,403,903	23,403,903	23,403,903	29,046,205	25,758,992	25,758,992	25,758,992	25,758,992	25,758,992	25,758,992	25,758,992
Patient ID	.	Patient 1	Patient 2	Patient 3	.	Index 1	Index 2	Index 3	Index 4	Index 5	Index 7	Index 8
Gender	male	female	female	female	male	female	male	female	male	female	male	female
CNVS	dup	del	del	dup	dup	dup	del	dup	dup	del	dup	del
Parental segregation	de novo	de novo	de novo	de novo	de novo	de novo	de novo	de novo	de novo	de novo	From mother	de novo
Speech delay	+	+	+	_	+	+	+	+	+	+	NA	+
Motor delay	+	+	+	+	+	+	+	+	+	+	+	+
Hypotonia	+	NA	NA	NA	NA	+	+	NA	+	+	NA	_
DD/ID	+(moderate–severe)	+	+	_	NA	+(mild–moderate)	+(mild–moderate)	+(moderate)	+(moderate)	+(mild–moderate)	_	+(moderate)
Heart defects	+(ASD)	+(complex)	+(TOF)	+(PVSD)	+(VSD)	_	_	_	_	_	+(PFO)	+(VSD)
Seizures	+	NA	NA	NA	+	NA	NA	NA	+	NA	NA	NA
Autistic features	_	NA	NA	_	NA	_	+	_	_	_	NA	NA
Behavioral troubles	+	NA	NA	NA	+	NA	+	NA	+	NA	NA	NA
Vision impairment	+	+	+	NA	+	+	+	+	+	+	NA	NA
Bulbous nasal tip	+	NA	+	NA	NA	NA	NA	NA	NA	NA	NA	NA
Cleft lip	+	_	_	_	_	_	_	_	_	_	_	_
Cupid‐bow upper lip	+	NA	NA	NA	NA	NA	NA	NA	NA	NA	NA	NA
Hearing impairment	+	NA	NA	NA	NA	NA	NA	NA	_	NA	+	NA

PMID	29,511,999	29,511,999	29,511,999	29,511,999	29,511,999	29,159,987	28,371,282	28,371,282	37,512,036	24,781,760	31,337,854	39,257,968	39,257,968
Patient ID	patient 1	patient 2	patient 3	patient 4	patient 24	Patient 1	patient 2	patient 3	.	patient 2	patient 34	F5S1	F8S1
Gender	female	male	male	male	female	female	female	female	male	male	female	female	male
CNVS	del	del	del	del	del	del	del	del	del	del	del	del	del
Parental segregation	NA	de novo	de novo	de novo	de novo	de novo	From mother	From mother	de novo	de novo	de novo	NA	de novo
Speech delay	+	+	+	+	+	+	NA	+	+	+	NA	NA	NA
Motor delay	+	+	+	+	+	+	+	+	_	+	NA	+	NA
Hypotonia	+	NA	+	+	+	+	NA	NA	NA	NA	NA	+	+
DD/ID	+	+	+	+	+	+(severe)	+(mild DD)	+	NA	NA	+	NA	NA
Heart defects	+(PFO)	NA	_	NA	_	NA	NA	NA	_	+(PFO)	+(ASD)	NA	NA
Seizures	_	_	_	_	_	NA	NA	NA	NA	NA	NA	_	_
Autistic features	_	+	NA	+	_	NA	NA	NA	NA	NA	NA	NA	NA
Behavioral troubles	NA	NA	NA	+	NA	+	NA	NA	NA	_	+	NA	NA
Vision impairment	+	NA	NA	NA	NA	NA	NA	NA	+	+	NA	+	_
Bulbous nasal tip	+	+	+	+	+	NA	NA	NA	NA	NA	NA	NA	NA
Cleft lip	_	_	_	_	_	_	NA	_	NA	_	_	NA	NA
Cupid‐bow upper lip	NA	+	+	+	+	NA	NA	NA	NA	NA	NA	NA	NA
Hearing impairment	NA	NA	NA	NA	NA	NA	NA	NA	_	_	NA	NA	NA

PMID	39,257,968	39,257,968	39,257,968	39,257,968	39,257,968	39,257,968	39,257,968	39,257,968	39,257,968
Patient ID	F8S2	F11S1	F18S1	F23S1	F28S1	F30S1	F36S1	F40S1	F43S1
Gender	male	female	female	male	male	male	male	male	male
CNVS	del	del	del	del	del	del	del	del	del
Parental segregation	de novo	de novo	NA	de novo	NA	NA	NA	de novo	de novo
Speech delay	NA	NA	NA	NA	NA	NA	NA	NA	NA
Motor delay	NA	NA	NA	NA	NA	NA	NA	NA	NA
Hypotonia	+	+	+	NA	+	_	+	+	+
DD/ID	NA	+	+	NA	NA	NA	NA	+	NA
Heart defects	NA	NA	NA	NA	NA	NA	NA	NA	NA
Seizures	+	_	_	_	_	_	_	_	_
Autistic features	NA	NA	NA	NA	NA	NA	NA	NA	NA
Behavioral troubles	NA	NA	NA	NA	NA	NA	NA	NA	NA
Vision impairment	_	+	+	+	+	+	+	_	+
Bulbous nasal tip	NA	NA	NA	NA	NA	NA	NA	NA	NA
Cleft lip	NA	NA	NA	NA	NA	NA	NA	NA	NA
Cupid‐bow upper lip	NA	NA	NA	NA	NA	NA	NA	NA	NA
Hearing impairment	NA	NA	NA	NA	NA	NA	NA	NA	NA

Among these, a pair of sisters carrying *MED13L* deletion variants (inherited from their mother) both exhibited motor delay and global developmental delay (DD). One male patient with a maternally inherited *MED13L* duplication variant had a congenital heart defect and motor delay, but he did not have an intellectual disability (ID).

Regarding ID/DD: among patients with *MED13L* duplication variants (including our reported case), 2 out of 6 had no ID/DD; among patients with deletion variants, all 17 evaluable patients had ID/DD (17/17). Regarding congenital heart defects: among duplication variants, 4 out of 7 patients with available cardiac data had a heart defect; among deletion variants, 6 out of 11 patients with available cardiac data had a heart defect. Only three patients presented with autistic features, all of whom carried deletion variants. All enrolled patients with *MED13L* variants exhibited characteristic facial dysmorphism. Common features included bulbous nasal tip (7/34), Cupid's bow upper lip (5/34), and hypotonic open mouth. Additional frequently observed features comprised deep philtrum, thin vermilion border, broad or depressed nasal bridge, and frontal bossing or bitemporal narrowing. (Further case information was provided in Table S1).

## 4 Discussion and Conclusion

*MED13L* was initially associated with TGA and ID, and three missense variants were discovered in TGA patients (Muncke et al. 2003). The original report describing three patients with *MED13L* copy‐number variants established *MED13L* haploinsufficiency syndrome. Studies have indicated that, in comparison to previously known missense variants, *MED13L* haploinsufficiency causes a distinct syndrome phenotype, with certain missense variants resulting in isolated heart defects. To date, only one patient has been reported with a *MED13L* duplication variant (also involving 11 other genes) inherited from his mother; his sister also carried the same duplication, and all three affected members had a mild phenotype (Adegbola et al. 2015). Only two patients with *MED13L* deletion variants were not de novo; they were sisters who inherited the same intragenic deletion from their mother. Both exhibited characteristic facial features and developmental manifestations, except that the older sister had premature craniosynostosis (Yamamoto et al. 2017). *MED13L* syndrome is rare. A total of 27 patients with *MED13L* copy‐number deletion variants and 6 patients with partial or complete *MED13L* duplication variants have been retrieved from the literature (including 3 with intragenic duplication and 3 with whole‐gene duplication). The present study adds one additional patient with a partial duplication (exons 8–16), bringing the total number of reported duplication cases to 7.

Our patient presented with clinical manifestations including language and motor delay, facial dysmorphism, and atrial septal defect, consistent with previously reported *MED13L*‐associated phenotypes (Cafiero et al. 2015; Adegbola et al. 2015; Yamamoto et al. 2017). Notably, while cleft lip has never been observed in prior cases, our patient is the first to exhibit this feature, underscoring the variable expressivity of the *MED13L*‐related phenotype.

*MED13L* plays a key role in transcriptional regulation during brain development, orchestrates cortical neurogenesis by priming the transcriptional activation of key developmental genes, including Neurod2, Sox5, Auts2, and Nfib (Li et al. 2025; Hamada et al. 2023). The patient in the present study had mild to severe global developmental delay, and among all collected cases, a significant proportion of *MED13L* variant carriers presented with ID/DD (4/6 of duplication cases and 17/17 of deletion cases), indicating that ID/DD is a widespread feature of *MED13L* syndrome, with a higher proportion in deletion carriers than in duplication carriers. We found that visual impairment is a common and important phenotype of *MED13L* syndrome, with a prevalence of up to 20/23. Manifestations include strabismus, myopia, hyperopia, upper eyelid coloboma or fissures, blepharoptosis, epicanthal folds, deeply set eyes, and lower eyelid inversion (Table S1). This phenotype was present in all five patients with duplications (5/5) and in the majority of those with deletions (15/18). A literature review of 33 patients with *MED13L* variants confirmed that the majority exhibited recognizable facial dysmorphism (Smol et al. 2018), which may overlap with features of 1p36 microdeletion syndrome (OMIM #607872) in some cases (Cafiero et al. 2015). The facial dysmorphism of our patient was characterized by a wide forehead, short palpebral fissures, a broad nasal bridge, a wide, open mouth with a large tongue, low set ears, and a biological block in the right ear, typical features of *MED13L* syndrome.

Our patient was unable to walk independently, did not imitate speech sounds, produced only non‐meaningful single sounds, had speech and motor delays, and exhibited no other severe behavioral problems. Among the collected cases, some patients exhibited behavioral problems. Two patients with duplication variants had repetitive behavior, attention deficit hyperactivity disorder (ADHD), agitation, restlessness, and excessive friendliness. Five patients with deletion variants presented with behavioral problems such as stereotypic movements, anxiety, aggressive behavior, attention deficit disorder, avoidance of eye contact (Table S1). Notably, both patients with aggressive behavior had deletion variants, suggesting a potential association between deletions and this behavioral phenotype; however, this observation requires confirmation in larger cohorts.

Among the six patients with *MED13L* duplication variants, the three with a whole‐gene duplication all had congenital heart defects (two VSD, one PFO, 3/6) (Asadollahi et al. 2013; Meng et al. 2017; Adegbola et al. 2015), whereas the three with multi‐exon partial duplications had no heart defect (3/6) (Adegbola et al. 2015). Our newly reported patient, who carries a *MED13L* duplication variant involving exons 8–16, also has a congenital heart defect (atrial septal defect, ASD), making this case particularly unusual. Only one patient with a deletion variant had Tetralogy of Fallot (TOF), and another patient with a deletion variant had a complex congenital heart defect including supracardial total anomalous pulmonary venous connection (TAPVC), pulmonary atresia with ventricular septal defect, and multifocal pulmonary perfusion (Asadollahi et al. 2013) (Table S1). Abnormal *MED13L* dosages have been shown to affect cardiac and neurodevelopment (Asadollahi et al. 2013). Both *MED13L* deletions and duplications may cause congenital heart disease according to our statistical analysis. The incidence of congenital heart defects did not differ significantly between patients with duplication variants (4/7) and those with deletion variants (6/11).

In contrast to patients with *MED13L* duplication variants, who showed no autistic features, a 50% prevalence of autism (3/6) was observed in patients harboring deletion variants (Adegbola et al. 2015; Smol et al. 2018). Seizures were observed in 3 of 7 individuals with duplications and 1 of 16 with deletions, representing a minority of patients in each group. The contrasting patterns in the occurrence of autistic features and seizures between these genotypes indicate variant type‐specific biases in phenotypic expression.

*MED13L* pathogenic missense variants are associated with varying degrees of clinical severity. Reports have found exon 15 variants (p.Pro866Leu and p.Pro869Ser) correlated with severe phenotypes, including epilepsy and severe motor impairment, whereas p.Gly1899Arg and p.Thr2162Met were associated with milder manifestations. These missense variants may disrupt *MED13L*'s interaction with the CDK8 kinase module, leading to functional deficits (Smol et al. 2025). Collectively, these findings provide insights into the differing incidence and severity of phenotypes between individuals with *MED13L* copy number duplications and those with deletions.

Previous suggestions that *MED13L* duplication variants result in milder phenotypes (e.g., congenital heart defects, developmental delay) than deletions (Asadollahi et al. 2013) are not substantiated by our collected data, particularly given the very limited sample size in prior studies (1 duplication vs. 2 deletions). In contrast, our findings provide stronger support that there are no significant differences in the incidence and severity of core phenotypes, including visual impairment and congenital heart disease, with the exception of ID/DD, for which the incidence is higher in deletion variants than in duplications. Aggressive behavior observed in two deletion cases may indicate a difference, but this finding requires more evidence. A notable exception is the different prevalence of autistic features and seizures between the two groups, which may suggest a genotype‐specific difference, though this observation requires further validation.

In conclusion, our report describes a novel, likely pathogenic heterozygous duplication variant in *MED13L*, highlighting phenotypic variability in *MED13L*‐related neurodevelopmental disorders, including the newly recognized feature of cleft lip. Furthermore, for high‐incidence phenotypes such as congenital heart defects, we observed no significant differences in prevalence or severity between duplication and deletion carriers. However, the higher rates of aggressive behavior, autistic features, and seizures in the deletion group (compared to duplication carriers) suggest potential mechanistic differences between the two variant types, warranting further clinical investigation.

## Author Contributions

Conceptualization: Yan Jiang, and Wenjing Zhao; data curation: Zhongqing Wang and Xin Chen; formal analysis: Zhongqing Wang, Xinchen, Ziyun Cui, Xinyue Qi, Li Gu, Yi Liu, Wenjing Zhao, Yan Jiang; writing – original draft: Zhongqing Wang, and Wenjing Zhao; writing – review and editing: Zhongqing Wang, Xinchen, Ziyun Cui, Xinyue Qi, Li Gu, Yi Liu, Wenjing Zhao, Yan Jiang; project administration: Yan Jiang and Wenjing Zhao.

## Funding

This work was supported by the National Natural Science Foundation of China [grant number 82160219], the Xingdian Talent Support Project of Yunnan Province [grant number XDYC‐QNRC‐2022‐0267], and the reserve Talents Project of Yunnan Provincial Health Commission [grant number H‐2024073].

## Ethics Statement

This study was approved by the Ethics Committee of the First People's Hospital of Yunnan Province. Written informed consent was obtained from participants (or their parent or legal guardian).

## Conflicts of Interest

The authors declare no conflicts of interest.

## Supporting information


**Table S1:** Detailed Genotype and Phenotype Correlations for MED13L.

## Data Availability

The data that support the findings of this study are available from the corresponding author upon reasonable request.
